# The Value of lncRNA NEAT1 as a Prognostic Factor for Survival of Cancer Outcome: A Meta-Analysis

**DOI:** 10.1038/s41598-017-10001-0

**Published:** 2017-10-12

**Authors:** Yunyuan Zhang, Limin Lun, Hui Li, Qing Wang, Jieru Lin, Runhua Tian, Huazheng Pan, Haiping Zhang, Xian Chen

**Affiliations:** Department of Clinical Laboratory, the Affiliated Hospital of Qingdao University, Qingdao, 266003 China

## Abstract

The present meta-analysis aimed to analyze available data to identify the prognostic role of NEAT1 in multiple carcinomas. A systematic search was performed by using several computerized databases from inception to June 7, 2017. The quantity of the publications was assessed according to MOOSE checklist. Pooled HRs with 95% CI was calculated to summarize the effect. A total of 12 studies with 3,262 cancer patients were pooled in the analysis to evaluate the prognostic value of NEAT1 in multiple tumors. High expression levels of NEAT1 were demonstrated to be associated with poor OS (HR = 1.71, 95%CI: 1.37–2.14, *P* < 0.001) and tumor progression (III/IV vs. I/II: HR 1.76, 95%CI: 1.40–2.21, *P* < 0.00001). Subgroup analysis showed that NEAT1 detection method (qRT-PCR) and sample size (more or less than 100) did not alter the predictive value of NEAT1 on OS in various cancers. According to the meta-regression results, the large heterogeneity of meta-analysis may be attributed to the differences of NEAT1 detection method. Furthermore, elevated NEAT1 expression significantly predicted lymph node metastasis (HR: 2.10, 95%CI: 1.32–3.33, *P* = 0.002) and distant metastasis (HR: 2.80, 95%CI: 1.60–4.91, *P* = 0.0003) respectively. The results indicate that NEAT1 expression level is a prognostic biomarker for OS and metastasis in general tumors.

## Introduction

Cancer is becoming the leading cause of mortality and morbidity for human health over the past decade^1^. According to 2014 Cancer Statistics, an estimated 1,665,540 new cancer cases and 585,720 cancer deaths are projected to occur in the United States^2^. To date, the mechanisms of oncogenesis and tumor progression have not been fully clarified and the widely used prognostic markers are still tumor node metastasis (TNM) stage and grade, high rates of recurrence, tumor size and drug resistance etc. Thus, many scientists are devoted to identify new potential biomarker for forecasting prognosis and predicting the therapeutic efficacy for cancer patients to improve their survival status.

Non-coding RNAs refer to different types of RNA which can not produce biologically meaningful RNA transcripts, usually including small interfering RNA (short interfering RNA, siRNA), PIWI-interacting RNA (piRNA), microRNA (miRNA) and long noncoding RNAs (lncRNAs) etc^3,4^. Recent articles have indicated that at least 95% of the human genome undergoes transcription to a huge array of RNA species and most of them are longer than 200 nucleotides^5^. Mounting evidence links expression changes of lncRNAs with complex diseases such as cancer. The dysregulation suggests that lncRNAs emerge as vital modulators in carcinomas and thus further emphasizes the potential role of lncRNAs in tumorigenesis and tumor progression^6,7^.

LncRNA nuclear-enriched abundant transcript 1 (NEAT1), which identified from the multiple endocrine neoplasia and functioned as an oncogene in many types of human cancers^8,9^, is an essential component of nuclear paraspeckles^10,11^. Recently, many observations indicate that the striking promoted expression pattern was associated with worse survival and high risk of cancer metastasis in patients with various carcinomas^12,13^. However, most individual studies assessing the implication of NEAT1 levels in cancer have been limited by small sample sizes and the controversial results. Therefore, a comprehensive meta-analysis of all eligible articles was performed to further evaluate the clinical feasibility of NEAT1 as a novel biomarker candidate as well as useful insights into the tumor clinicopathological features.

## Material and Methods

### Search strategy and Literature selection

We conducted a comprehensive search of PubMed, Embase, Cochrane Library, ScienceDirect, BioMed Central, Springer, ISI Web of Knowledge, together with three Chinese databases: China National Knowledge Internet (CNKI), Wanfang and Weipu databases to identify potential eligible studies published before June 7, 2017. The following keywords were used for searching: (“long noncoding RNA” OR “lnc RNA” OR “nuclear-enriched abundant transcript 1” OR “NEAT1”) AND (“cancer” OR “carcinoma” OR “tumor” OR “neoplasm”) AND “prognosis” or “prognostic” or “survival” or “metastasis”). The reference lists of primary literatures were manually searched for additional relevant articles. All available articles written in English that investigated the expression of NEAT1 and the prognosis of OS were included.

### Inclusion and exclusion criteria

Inclusion criteria are as following: (1) Articles investigating the expression pattern of NEAT1 in any malignant tumor; (2) Definite diagnosis or histopathology confirmed for patients with cancer; (3) Sufficient data for the computation of hazard ratios (HR) and corresponding 95% confidence intervals (CI) to construct the 2 × 2 contingency Table.

Exclusion criteria are as following: (1) Studies investigating the molecular structure and functions of NEAT1 or literatures not pertinent to the NEAT1; (2) Studies of non dichotomous NEAT1 expression and absence of survival outcome; (3) Duplicate publications as well as multiple duplicate data in the different works, excluding earlier and smaller sample data; (4) Correspondences, animal experiments, letters, editorials, expert opinions, talks, reviews and case reports without original data.

### Data extraction and Quality Assessment

Two investigators (XC and YYZ) extracted all the essential information from identified articles independently. According to the inclusion and exclusion criteria, the following information from each enrolled study was extracted: (1) first authors, publication year, study population, patients size, tumor type, follow-up time; (2) NEAT1 assessment method and specimen; (3) HR and their 95% CI of NEAT1 value for overall survival, patient number for TNM state and progression, distant metastasis or lymph node metastasis. HRs were preferred by multivariate analysis if the eligible studies provided both univariate and multivariate analysis as the multivariate values had higher precision on interpreting confounding factors. If any essential information were not available from the original article, best efforts were made to contact the corresponding author to obtain the missing data. If only Kaplan–Meier curves were provided in some studies, the survival rates were extracted from the graphical survival plots and the calculated HR and 95% CI was determined as the published methods^14,15^. As shown in supplementary materials (Table S1), all the included publications were evaluated based on the critical checklist of the Dutch Cochrane Centre proposed by MOOSE.

### Statistical analysis

The present analyses were performed using Stata SE12.0 (Stata Corporation) and RevMan5.3 software. The impact of NEAT1 expression on overall survival, metastasis, TNM stage and progression was described as Hazard ratio (HR) and corresponding 95% CIs. The combined effect size (ES) was considered as HR and should be statistically significant when the 95% CI did not overlap with 1. Heterogeneity across the enrolled studies was quantified with the *I* ^2^ statistics. The random-effects model was conducted to analyze the relationship between NEAT1 expression and clinical outcomes when calculated *I* ^2^ > 30%. The suspected factors for heterogeneity were investigated using meta-regression models. Probable publication bias was displayed by constructing a funnel plot or conducting Begg’s bias indicator test. P values < 0.05 was considered statistically significant.

## Results

### Included literatures

**Figure 1:**
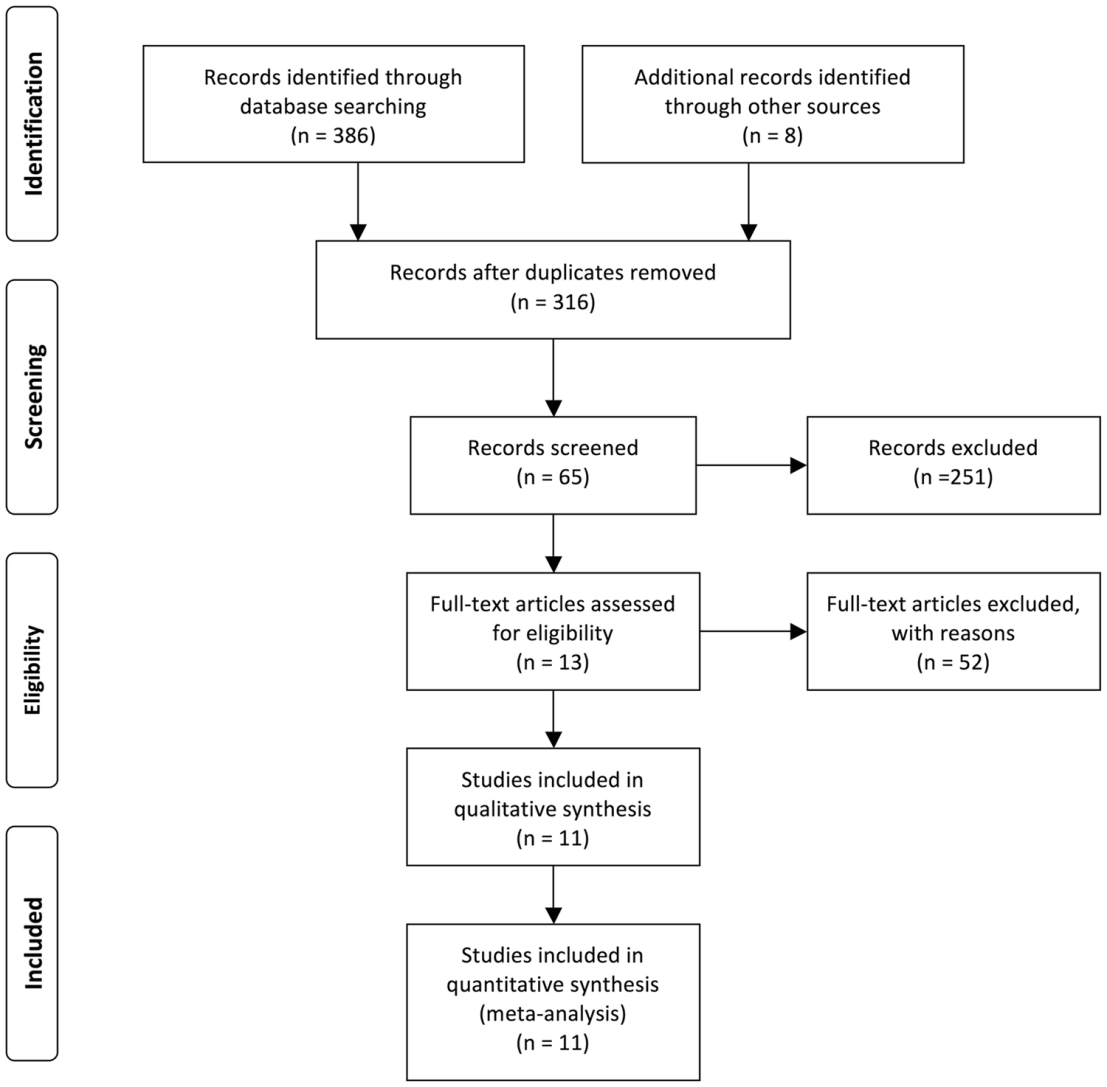
Flow diagram of the study search and selection process.

A total of 394 studies were initially identified as appropriate from electronic database search. After duplicate articles removed, 316 articles were left and these articles were then screened. After carefully screening the titles and abstracts, 251 articles were excluded and 66 articles were further reviewed of the full texts. 54 studies were then excluded because NEAT1 was not a dichotomic variable in the original studies. As shown in Fig. 1, the selection process with specification was presented by a flow diagram. Ultimately, the present meta-analysis was conducted for the remaining 12 articles.

### Characteristics of the enrolled studies

The main features of the 12 enrolled articles are summarized in Table 1 ^16–27^. These studies were published between 2014 and 2016 with sample sizes ranging from 57 to 2,000. All of the 3,262 patients were divided into two groups (high and low expression of NEAT1) according to the NEAT1 measurement results. Eleven of twelve studies were from China and the patients were ten types of carcinomas, including glioma, ovarian cancer, endometrial endometrioid adenocarcinoma, breast cancer, colorectal cancer, bladder cancer, nasopharyngeal carcinoma, gastric cancer, esophageal squamous cell carcinoma and non-small cell lung cancer. Of note, the median value was selected as the cut-off value in most articles.

**Table 1:** Summary of the twelve included studies.

Study	Origin of population	Study design	Disease	N	Stage	NEAT1 assay	Survival analysis	Metastasis analysis	Hazard ratios	Follow-up Months
Choudhry^16^	UK and Canada	R	BC	2000	NA	microarray detection	OS	NA	HR/K-M	144
Chen^17^	China	R	ESCC	96	I-II/III-IV	qRT-PCR	OS	LNM/DM	HR/K-M	80
He^18^	China	R	Glioma	94	I-II/III-IV	qRT-PCR	OS	NA	HR/K-M	80
Li^19^	China	R	CC	239	I-II/III-IV	qRT-PCR	OS, DFS	LNM/DM	HR/K-M	60
Pan^20^	China	R	NSCLC	57	I-II/III-IV	qRT-PCR	OS	NA	K-M	50
Chen^21^	China	R	OC	149	I-II/III-IV	qRT-PCR	OS	DM	HR/K-M	70
Fu^22^	China	R	GC	140	I-II/III-IV	qRT-PCR	OS	LNM/DM	HR/K-M	96
Lu^23^	China	R	NC	131	I-II/III-IV	qRT-PCR	OS	NA	K-M	60
Sun^24^	China	R	NSCLC	96	0-I/II-IV	qRT-PCR	OS	LNM	K-M	40
Chen^25^	China	R	BLC	65	0-I/II-IV	qRT-PCR	NA	LNM	NA	NA
Hu^26^	China	R	NSCLC	120	I-II/III-IV	qRT-PCR	NA	NA	NA	NA
Li^27^	China	R	EEA	75	I-II/III-IV	qRT-PCR	NA	LNM	NA	NA

Study design is described as retrospective (R); BC, Breast Cancer; ESCC, Esophageal Squamous Cell Carcinoma; GC, Gastric Cancer; CC, Colorectal Cancer; NC, Nasopharyngeal Carcinoma; BLC, Bladder Cancer; OC, Ovarian cancer; EEA,endometrial endometrioid adenocarcinoma; DM, distant Metastasis; LNM, Lymph Node Metastasis.

### NEAT1 and main outcome

**Figure 2:**
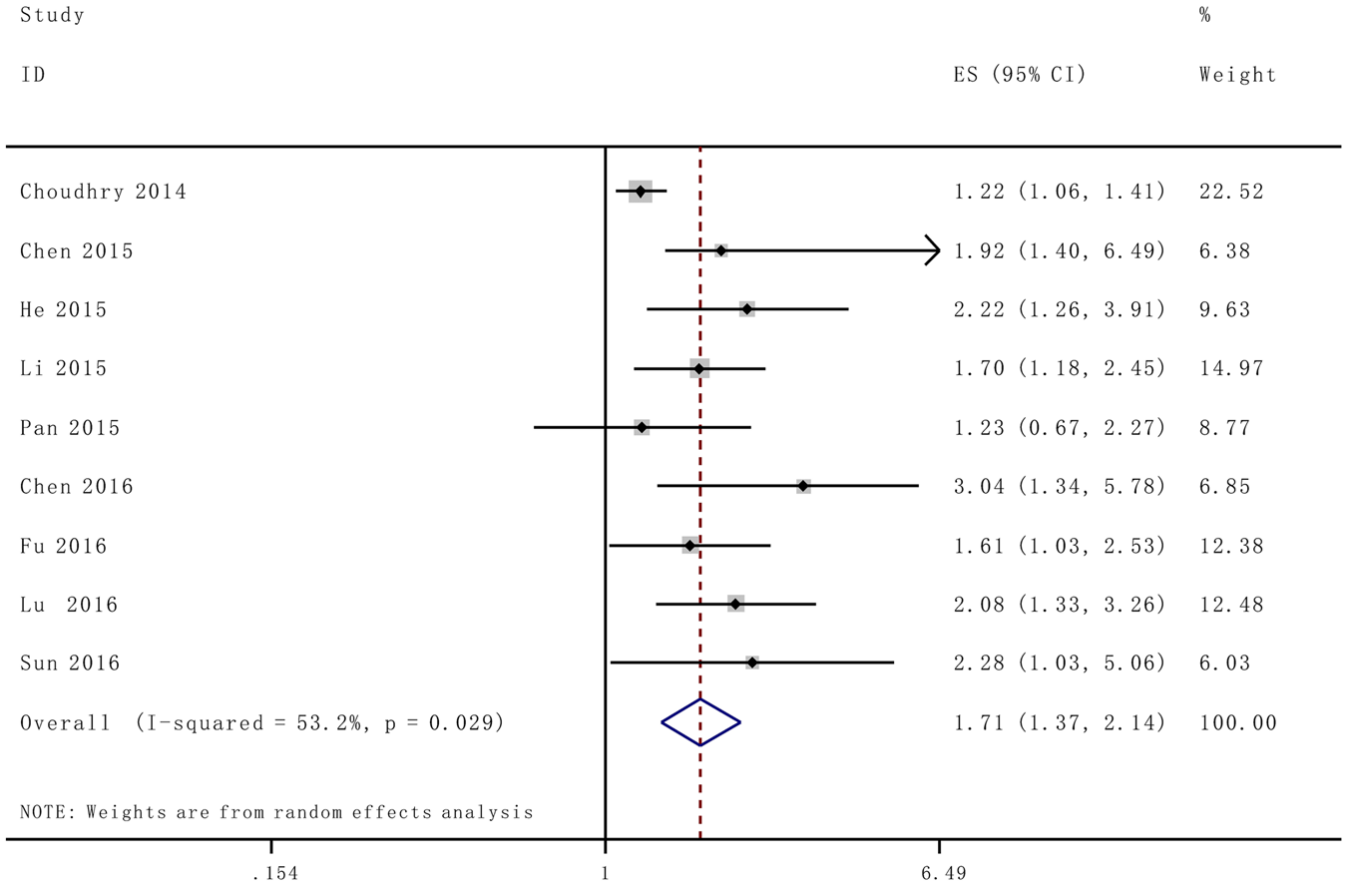
Forest plot for the association between NEAT1 expression with overall survival (OS).

The random effects model was used to analysis the pooled HR and its 95% CI because obvious heterogeneity was detected among those 9 studies which involved in OS analysis (*I* ^2^ = 59%). According to meta result in multivariate analysis, enforced NEAT1 expression was predictive of unfavorable OS in various carcinomas (HR = 1.79, 95% CI: 1.40–2.31, *P* = 0.000) (Fig. 2).

**Figure 3:**
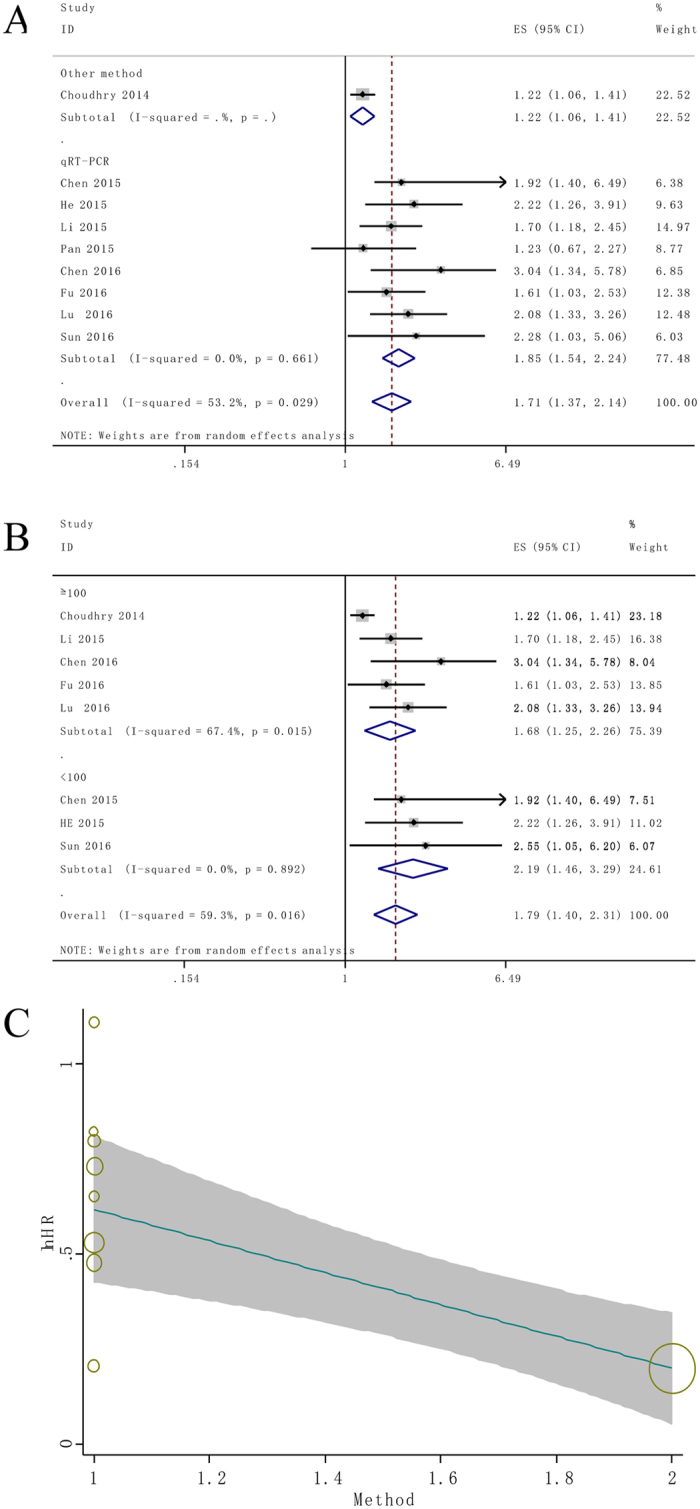
Stratified analyses and meta-regression analysis for the association between NEAT1 expression with overall survival (OS). (**A**) Subgroup analysis of HRs of OS by factor of detection method. (**B**) Subgroup analysis of HRs of OS by factor of sample size. (**C**) Meta regression analysis for assessment of the heterogeneity scores. Variable of suspected to heterogeneity was detection method.

Afterwards the stratified analyses and meta-regression models were performed by factor of NEAT1 detection method and sample size to analyze the possible sources of the heterogeneity (Table 2). The results from stratified analyses suggested that different detection methods and sample size (more or less than 100) do not alter the predictive value of NEAT1 on the OS for all involved cancers. No heterogeneity (*I* ^2^ = 0%) was detected in qRT-PCR subgroup analysis. Subsequently, meta-regression results revealed that the detection method was a significant factor for the heterogeneity of the primary results (*P* = 0.01) (Fig. 3).

**Table 2:** Subgroup analysis of the pooled HRs of overall survival with over-expressed NEAT1 in patients with cancer.

Subgroup analysis	No. of studies	No.of patients	Pooled HR (95% CI)		Heterogeneity	
			Fix/Random	*p* Value	I^2^(%)	*p* Value
Sample size						
≥100	5	2659	1.61 (1.24, 2.08)	<0.001	59.7	0.030
<100	4	343	2.15 (1.45, 3.19)	<0.001	0.0	0.943
Type of methods						
qRT-PCR	8	1002	1.85 (1.54, 2.24)	<0.001	0.0	0.661
Other methods	1	2000	1.22 (1.06, 1.41)	0.006	—	—

**Figure 4:**
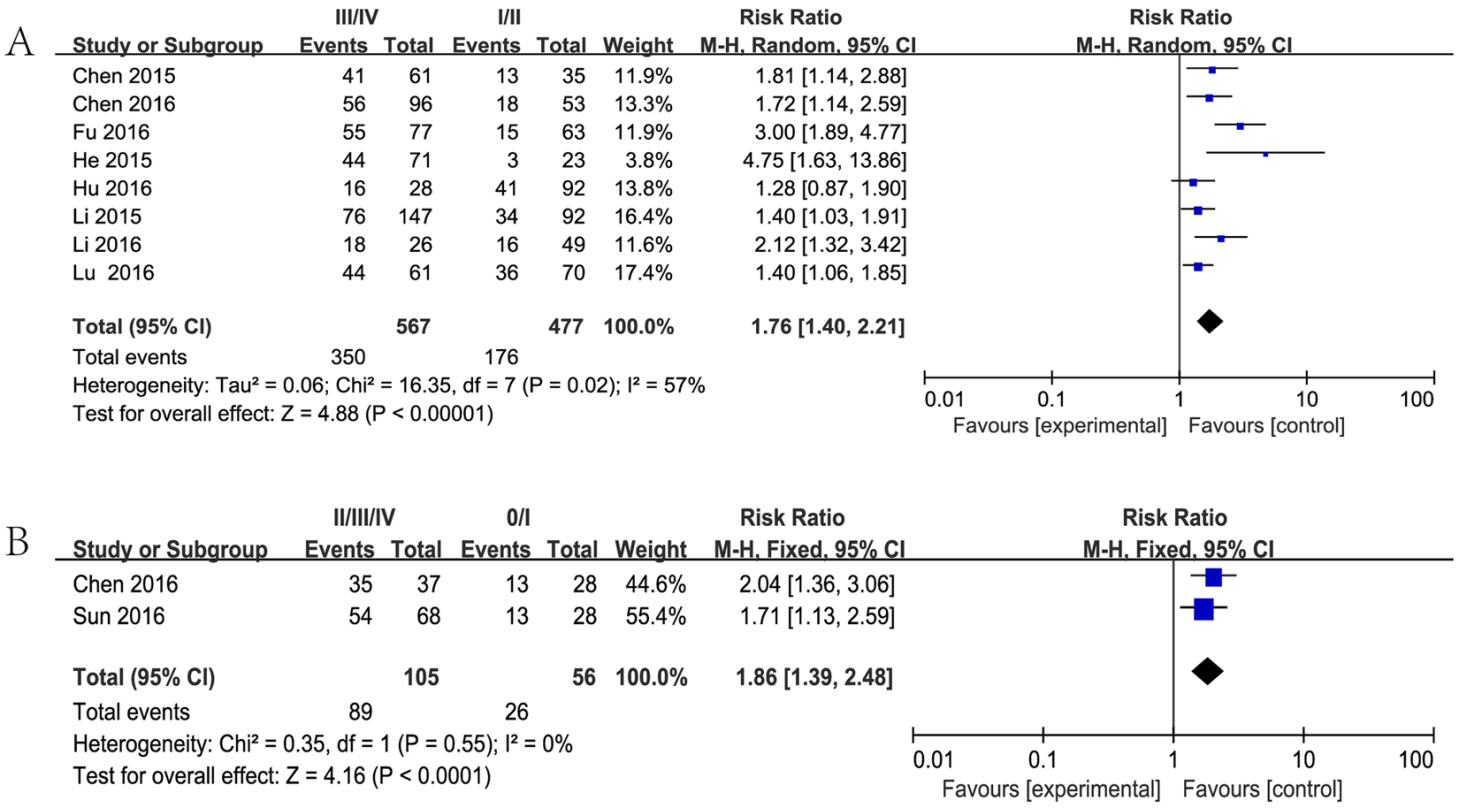
Forest plot for the association between NEAT1 expression with TNM stage (III/IV vs. I/II (**A**) and II/III/IV vs. 0/I (**B**)).

Increased NEAT1 expression was found to be moderately associated with tumor TNM stage and progression (III/IV vs. I/II: HR 1.76, 95%CI: 1.40–2.21, *P* < 0.00001 or II/III/IV vs. 0/I: HR 1.86, 95%CI: 1.39–2.48, *P* < 0.0001) (Fig. 4).

Overall, the pooled HRs revealed that NEAT1 expression might be served as a prognostic biomarker in various types of cancers.

### NEAT1 and metastasis

**Figure 5:**
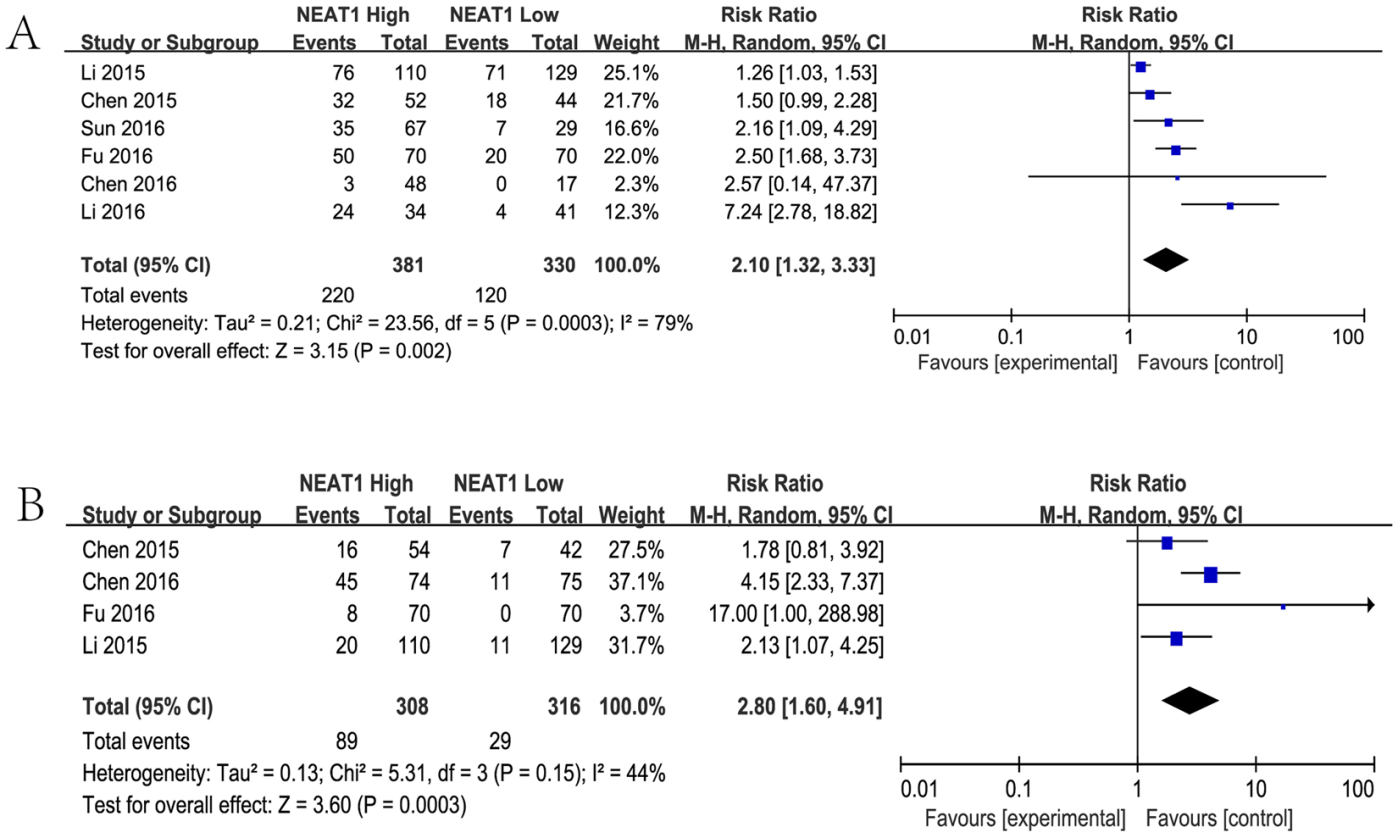
Forest plot for the association between NEAT1 expression with lymph node metastasis (**A**) and distant metastasis (**B**).

The characteristics of the involved studies which evaluating the association between NEAT1 levels and cancer metastasis were summarized in Fig. 5. A random model was performed to calculate the pooled HR and its 95% CI because there appeared to have heterogeneity between lymph node metastasis (*I* ^2^ = 79%) and distance metastasis (*I* ^2^ = 44%). Elevated NEAT1 expression significantly predicted lymph node metastasis (HR: 2.10, 95% CI: 1.32–3.33, *P* = 0.002) and distant metastasis (HR: 2.80, 95% CI: 1.60–4.91, *P* = 0.0003) respectively.

### Publication bias

**Figure 6:**
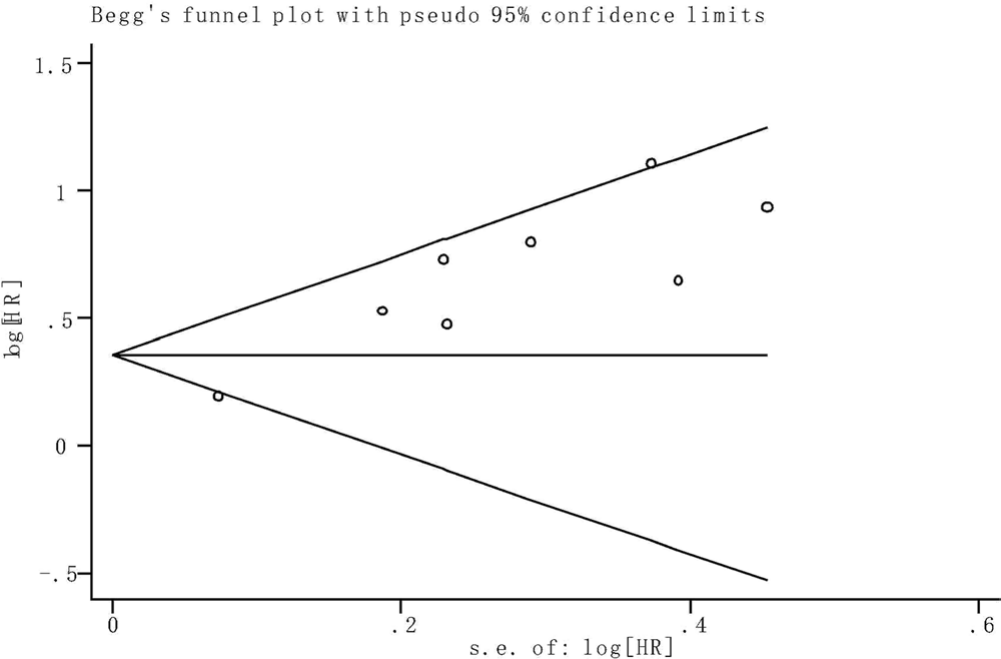
Funnel plot of the publication bias for overall survival.

The potential publication bias of the present meta-analysis was evaluated by a funnel plot and Begg’s indicator test. No evidence of publication bias was detected in the multivariate analysis of OS (*P* = 0.466) of all enrolled articles. The shape of the funnel plot did not reveal any evidence of obvious asymmetry (Fig. 6).

### Sensitivity analysis

**Figure 7:**
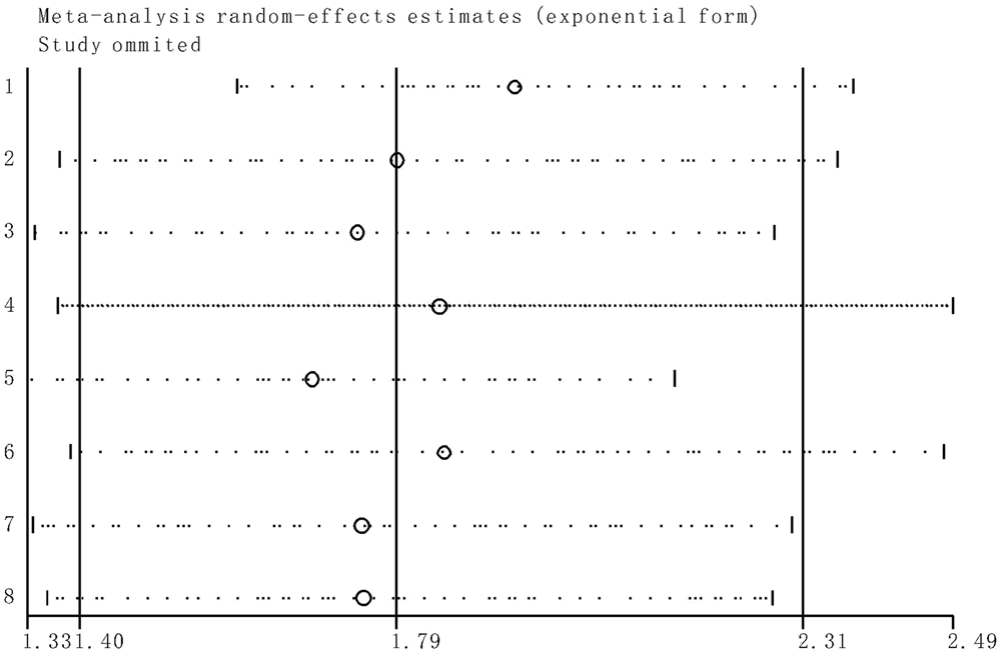
Sensitivity analyses of studies concerning NEAT1 and overall survival.

The stability of the crude results was evaluated by sensitivity analysis. The results suggested that the conclusions are stable because the pooled HR was not significantly affected by the exclusion of any single study (Fig. 7).

## Discussion

LncRNAs were defined as transcriptional noise in the past decades because most of them produced from intergenic and intronic regions of the genome and lack protein coding capability. In recent years, tremendous contributions were made by scientists to the discovery that lncRNAs regulate the target gene expression and act as oncogene or tumor suppressor^28,29^. With the rapid development of high-throughput genome-wide analysis technology, lncRNAs have been proposed as promising biomarkers for early detection and accurate prognosis for various carcinomas^30,31^.

NEAT1 was originally identified as a critical component of the paraspeckle structure which has been demonstrated to be involved in the transcriptional regulation of target gene expression^8,10,32^. It was also demonstrated that NEAT1 could respond to cellular cues and ligand signaling in a manner of the coding transcriptome, indicating a role for NEAT1 beyond its interaction with paraspeckles^8^. Recent studies manifested that NEAT1 contribute to the tumor progression through its regulation of diverse cellular processes, including migration, invasion, proliferation, differentiation, and apoptosis^27^. Li *et al*.^32^ found that Knockdown of NEAT1 in ovarian cancer cell lines inhibited cell proliferation through impeded the G1 cells from entering the S phase. In line with these finding, silencing of NEAT1 significantly induced the apoptosis of ovarian cancer cells through down-regulate the expression of Bcl-2, and up-regulate the expression of Bax, Caspase 3 and Caspase 9. Additionally, NEAT1 has been demonstrated to function as a competing endogenous RNA (ceRNA) in NSCLC that binds to and reduces the expression of a number of miRNAs. The reduction of miR-377–3p and let-7e leads to tumor progression through the miR-377–3p/E2F3 and let-7e/NRAS signaling pathway^22,33^.

Emerging evidence is encouraging that high expression of NEAT1 serves as a convinced poor prognosis in several types of cancers, such as glioma, ovarian cancer, endometrial endometrioid adenocarcinoma, breast cancer, colorectal cancer, bladder cancer, nasopharyngeal carcinoma, gastric cancer, esophageal squamous cell carcinoma, non-small cell lung cancer and so on. In the current study, we performed the comprehensive and detailed meta-analysis to investigate the clinical prognostic role of NEAT1 with a variety of carcinomas. Twelve studies including 3,262 patients were pooled in this study, and the results indicated that elevated NEAT1 expression was significantly correlated with poor prognosis, progression, LNM and DM in patients with various types of cancer. The analysis showed a pooled HR was 1.71 (95%CI: 1.37–2.14, *P* < 0.001), 1.76 (95%CI: 1.40–2.21, *P* < 0.00001), 2.10 (95% CI: 1.32–3.33, *P* = 0.002) and 2.80 (95% CI: 1.60–4.91, *P* = 0.0003) for OS, TNM stage (III/IV vs. I/II), LNM and DM respectively.

Recently, a quantitative analysis also indicated that NEAT1 over-expression predicted worse outcome in several cancers with the pooled HR being 1.53 (95% CI: 1.36–1.71, *p* < 0.001) for OS^33^. Distinct from earlier analysis, the current work paid substantial attention to the details of study design and data extracting in quality assessment. In order to guarantee the reliability of pooled results and consequently avoid the error generated from data extracting processes, HR values provided by primary research articles were extracted with priority^18,21^. Besides, the current analysis focuses not only on published qRT-PCR results, but also available gene expression data^16^. The improvement avoids the selection bias because Michelhaugh *et al*. has demonstrated the affymetrix microarrays do reliably capture the expression of NEAT1^34^. In addition, we set exacting inclusion and exclusion criteria to ensure the quality of involved studies and thus the reliability of pooled results. For instance, two studies that measured NEAT1 variants levels but not total NEAT1 expression were excluded^35,36^, although both studies reported that high levels of NEAT1 variants were related with poor survival in tumor, which was consistent with our conclusion in the present analysis. By these means, we fulfilled a comprehensive research and achieved objective and unbiased conclusions.

Nevertheless, it should be noted that there are several limitations of this study should be discussed. First, some of the HRs were calculated by reconstructing survival curves rather than directly obtained from the primary studies. Second, significant heterogeneity was observed in the analysis when we pooled HRs. Under this condition, we used the random effects model to pool the data. As a matter of fact, meta-regression results suggested that the large heterogeneity of meta-analysis may be attributed to the differences of NEAT1 detection method. Finally, cut off definition were different in each study, which might weaken the reliability of our conclusion.

In aggregate, even some limitations mentioned above, it is preliminarily concluded that promoted NEAT1 may be considered as a credible unfavorable prognostic factor in human cancers. In the future, well designed larger-sample studies and better design studies will be necessary to verify and strengthen the prognostic role of NEAT1 in neoplasm patients.

## Electronic supplementary material

Supplementary Table
