# Supplementary material for: The Value of lncRNA NEAT1 as a Prognostic Factor for Survival of Cancer Outcome: A Meta-Analysis
Source: Sci Rep. 2017 Oct 12;7:13080. doi: 10.1038/s41598-017-10001-0 (PMC5638961; doi:10.1038/s41598-017-10001-0)
Supplement: Supplementary file 1 — Supplementary Table [file 41598_2017_10001_MOESM1_ESM.pdf]

The Value of lncRNA NEAT1 as a Prognostic Factor for Survival of Cancer Outcome: A Meta-Analysis

Xian Chen[1#], Jieru Lin[2#], Limin Lun[1], Runhua Tian[1], Huazheng Pan[1],
Haiping Zhang[1] and Yunyuan Zhang[1*]

[1]Department of Clinical Laboratory, the Affiliated Hospital of Qingdao University, Qingdao 266003, China
2Department of Respiratory and Critical Care Medicine, Guizhou Provincial People's Hospital, Guiyang, PR China.

Running title: A meta-analysis for NEAT1 as a Prognostic Factor in various carcinomas.

# These authors contribute equally to the article.

*Corresponding author

Correspondence to: Dr Yunyuan Zhang, Department of Clinical Laboratory, the Affiliated Hospital of Qingdao University, Qingdao 266003, China

E-mail: hellozyycool@163.com; Tel: +86-18661803058 Fax: +86-0532-82911229

Table S1. MOOSE checklist

**Reporting of background should include**	
Problem definition	Background (Page 3)
Hypothesis statement	Background (Page 3)
Description of study outcome(s)	OS, TNM,LNM,DM
Type of exposure or intervention used	Various cancer (Page 3)
Type of study designs used	Meta-analysis (Page 3)
Study population	Global (Page 3)
**Reporting of search strategy should include**	
Qualifications of searchers (eg, librarians and investigators)	Investigator(Page 4)
Search strategy, including time period included in the synthesis and keywords	Search strategy and selection criteria (Page 4)
Effort to include all available studies, including contact with authors	We contact authors and searched reference lists and citations (Page 4)
Databases and registries searched	Methods (Page 4)
Search software used, name and version, including special features used (eg, explosion)	IE 10
Use of hand searching (eg, reference lists of obtained articles)	Search strategy and selection criteria (Page 4)
List of citations located and those excluded, including justification	Flow diagram in Figure 1. (Page 5)
Method of addressing articles published in languages other than English	Search strategy and selection criteria (Page 4)
Method of handling abstracts and unpublished studies	Method (Page 4)
Description of any contact with authors	Method (Page 4)
**Reporting of methods should include**	
Description of relevance or appropriateness of studies assembled for assessing the hypothesis to be tested	Method (Page 4)
Rationale for the selection and coding of data (eg, sound clinical principles or convenience)	Methods (Page 4)
Documentation of how data were classified and coded (eg, multiple raters, blinding, and interrater reliability)	Methods (Page 4)
Assessment of confounding (eg, comparability of cases and controls in studies where appropriate)	Methods (Page 4)
Assessment of study quality, including blinding of quality assessors; stratification or regression on possible predictors of study results	Methods (Page 4)
Assessment of heterogeneity	Methods (Page 5)

Description of statistical methods (eg, complete description of fixed or random effects models, justification of whether the chosen models account for predictors of study results, dose-response models, or cumulative meta-analysis) in sufficient detail to be replicated	Methods (Page 5)
Provision of appropriate tables and graphics	Methods and Results (Page 5-6)
**Reporting of results should include**	
Graphic summarizing individual study estimates and overall estimate	Figure 2, 3, 4 and 5
Table giving descriptive information for each study included	Table 1
Results of sensitivity testing (eg, subgroup analysis)	Sensitivity analysis (Page 7)
Indication of statistical uncertainty of findings	Discussion (Page 6)
**Reporting of discussion should include**	
Quantitative assessment of bias (eg, publication bias)	Discussion (Page 7)
Justification for exclusion (eg, exclusion of non–English-language citations)	Discussion (Page 8)
Assessment of quality of included studies	Discussion (Page 8)
**Reporting of conclusions should include**	
Consideration of alternative explanations for observed results	Discussion (Page 8)
Generalisation of the conclusions (ie, appropriate for the data presented and within the domain of the literature review)	Discussion (Page 8)
Guidelines for future research	Discussion (Page 9)
Disclosure of funding source	Grant Support (Page 9)
